# The concept of justifiable healthcare and how big data can help us to achieve it

**DOI:** 10.1186/s12911-021-01444-7

**Published:** 2021-03-06

**Authors:** Wim van Biesen, Catherine Van Der Straeten, Sigrid Sterckx, Johan Steen, Lisa Diependaele, Johan Decruyenaere

**Affiliations:** 1 Renal Division, 0K12 IA, Ghent University Hospital, Corneel Heymanslaan 10, 9000 Gent, Belgium; 2 Consortium for Justifiable Healthcare, Ghent University Hospital, Ghent, Belgium; 3 Health, Innovation and Research Institute, Ghent University Hospital, Ghent, Belgium; 4 Bioethics Institute Ghent, Department of Philosophy and Moral Sciences, Ghent University, Ghent, Belgium; 5 Department of Intensive Care, Ghent University Hospital, Ghent, Belgium

## Abstract

Over the last decades, the face of health care has changed dramatically, with big improvements in what is technically feasible. However, there are indicators that the current approach to evaluating evidence in health care is not holistic and hence in the long run, health care will not be sustainable. New conceptual and normative frameworks for the evaluation of health care need to be developed and investigated. The current paper presents a novel framework of justifiable health care and explores how the use of artificial intelligence and big data can contribute to achieving the goals of this framework.

## Background

Every patient expects to receive the best care for their problem. Every physician aims to provide the best care for their patient. Every public policymaker and politician should strive to make the best care available to all members of society. All these appear straightforward aspirations, but bringing them into reality rapidly turns out to be quite challenging. The face of healthcare has changed substantially over the last 2 decades, and continues to do so at exponential speed. Fuelled by digitalization and artificial intelligence techniques, new interventions and diagnostics are being developed, stretching expectations on what can be achieved in healthcare to the limits of human imagination. The expansion of tools in the field of genomics in the last decade has enabled not only the analysis but also the modification of the human genome [1]. At the same time, healthcare costs are surging rapidly, and health is increasingly considered as a consumption good, and thus subject to the workings of the free market [2, 3].

In such a setting, what exactly *is* the best care for a certain condition? How can we define or know whether A is a better intervention than B, who defines this, and on the basis of which criteria? Most would argue that this can best be found out via randomized controlled trials (RCTs), ideally several of them aggregated in rigorously performed systematic reviews [4, 36]. RCTs have the great advantage that, when performed properly, they can theoretically directly reveal causal relations between an intervention and the effect size of an outcome. Therefore, most would consider them as the highest rank of evidence. However, it might be unclear whether the best intervention should be the *same* for *all* patients with a particular condition? A further question is in which conditions can A be an acceptable treatment, despite being less optimal than B? Different answers may arise due to various reasons. Patients may have different preferences, both with regard to the intervention, as to which outcome they prefer. For example, some patients on hemodialysis prefer a tunnelled catheter over an arteriovenous fistula [5, 6], although the latter are associated with better outcomes in observational studies. Furthermore, the cost of A can be exuberantly higher than the cost of B, while the difference in outcome is minimal. The management of hyperkalaemia can be done with (cheap) resins or (expensive) patiromer, but evidence to compare one head to head to the other is currently lacking.

What if according to the physician A is better than B, but the patient still wants B? What if intervention A is best for disease X, but also very expensive and thus potentially ruling out the reimbursement of intervention B for disease Y (sustainability, fairness, opportunity costs) [7]? What if an intervention is good for an individual but not for society, for example the unrestricted use of broad spectrum antibiotics? What if available information is apparently conflicting or contradictory? What if patients are lured into treatments that have little or no effect (for example by direct to consumer marketing)?

In this paper, we will first elaborate the concept of *justifiable healthcare* as a means to strive for evidence based, efficient, just and sustainable healthcare. We will first explore the different steps of justifiable healthcare evaluation, why they are necessary and what the pitfalls and bottlenecks are. More importantly, in the second part of the paper, we will explore how the use of *big data* and *artificial intelligence* technology may be helpful in tackling some of the problems in achieving such justifiable healthcare, and how it might in contrast generate additional hurdles and pitfalls [8].

Although a universally accepted definition of big data does not exist, most definitions refer to an approach which allows for an enormous increase in access to, and automated use of, datapoints, and this at a speed beyond the ability of classical database software. Frequently mentioned additional attributes of big data are the variety of the available data and data sources that can be used and aggregated. Using algorithms (artificial intelligence), these datapoints can be turned into new information and knowledge. Routinely collected health data (RCD, sometimes also denoted as Real World Data), for example obtained from electronic health records or insurance claims, but also from wearables or apps, are increasingly used for biomedical purposes. This is primarily motivated by the expectation that revealing valuable information hidden within these data will improve medical decision-making, assist regulatory approval, and reduce costs. However, this potential utility inherently hinges on data quality which is often compromised by missing, miscoded or erroneous entries, maladapted data capture incentives (upcoding to maximize charges) or server shutdowns [9]. For example, it has been debated whether the improved outcome of acute kidney injury over the last decades is a real improvement or just a bias induced by a creeping of the definition in administrative datasets [10]. Moreover, setting up collaborative data networks poses additional challenges related to interoperability and data harmonization [11]. Data coming from different sources should first be harmonized and aligned to a common data model before they can be used for analysis or knowledge generation. Once this step is taken the data should be tested for three main data quality criteria: conformance, completeness and plausibility [12]. This is rarely a swift process and many real life big data applications fail in this regard, leading to biased conclusions [13, 14].

## Introducing the concept of justifiable healthcare

The *best care* is a fragile concept to handle, as it is very difficult to define “best”, or even to determine whose perspective should be taken when defining “best”: what is best from the perspective of A in situation X may be not best from perspective B in situation Y, where A and B can be potential stakeholders involved in healthcare (patients, family, physicians, policymakers, healthcare providers, society, industry..) and X and Y represent different conditions and settings. Therefore, rather than considering *best care*, we introduce the new concept of *justifiable healthcare*.

**Fig. 1:**
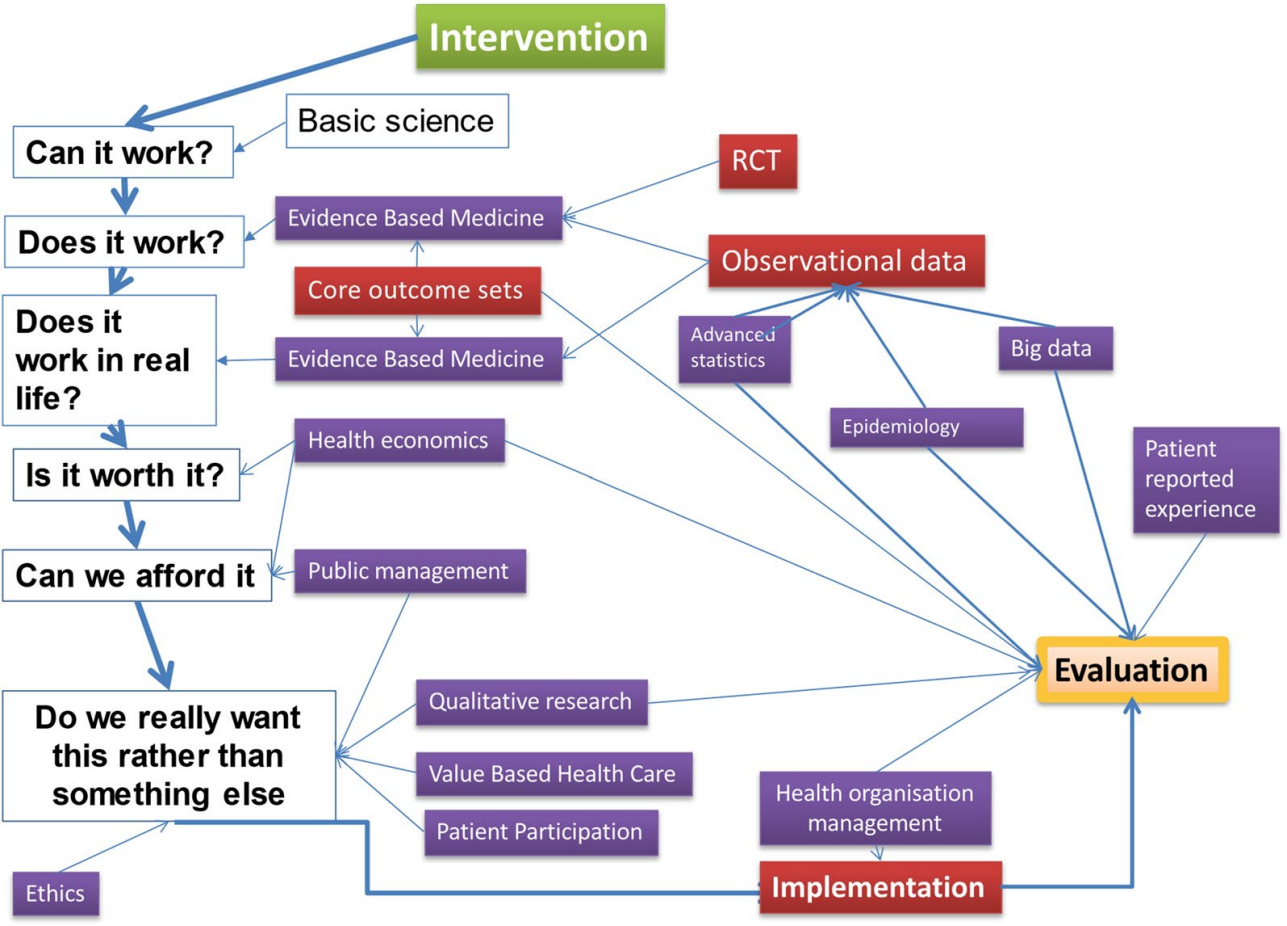
Flow chart of justifiable healthcare

For the purposes of ascertaining whether a certain health intervention is *justifiable*, we propose a holistic approach, informed by different perspectives and disciplines, following predefined consecutive steps (Fig. 1). Justifiable healthcare differs from good/best care, as it implicitly requires the underlying reasoning and argumentation to be made ***transparent*** to all stakeholders, and the decision to be based on a ***systematic*** and ***holistic*** approach. This differs from the frequently adopted paternalistic approach, where “best” is largely defined based on purely *biological* and *technical* aspects of care, and where the decision is taken in isolation, without consideration of its societal impact, psycho-social aspects or what patient needs and values. Indeed, justifiable healthcare implies *transparency* and *evaluation*, both at the micro-level of the interaction with the patient and at the macro-level of society, for example for drug registration or reimbursement (Table 1).

**Table 1:** Evidence generation within the justifiable healthcare model

Step	Tool/method	Epistemologic aim	Pitfalls/problems	Role for big data/AI
**Step 1: Evidence based medicine**				
Generate hypothesis	Physiology Pathophysiology Anatomy Biology Pathogenesis	*Description:* Describe biological processes in detail *Causal Inference:* Enhance mechanistic understanding of basic biological processes and how they can be influenced	Information overload Transportability/generalizability Incorrect analytical approach	Pattern recognition Classification Clustering Searching and Aggregating all already existing knowledge Prediction of mode of action of different molecular structures as derived from existing knowledge Propose alternative structures based on existing molecular knowledge
	Epidemiology	*Description:* Describe population in detail for given conditions and outcomes: prevalence, incidence, risk factors, associated factors *Prediction:* Predict relevant outcomes based on a given set of covariates for a given population (association) or an individual from that population (risk prediction or prognosis) *Causal Inference:* Enhance understanding of causal effect association between a covariate and an outcome in a given population	Data quality Transportability/generalizability Incorrect analytical approach (remaining confounding and bias; incorrect causal inference)	Pattern recognition Classification Clustering Searching and Aggregating all existing knowledge Prognostication Trial emulation Trial simulation Causal inference techniques Dynamic Decision Problems
Proof of concept	(small) phase 1 and 2 trials	*Description:* describe properties of population *Prediction:* Predict relevant outcomes based on a given set of covariates for a given population (association) or an individual from that population (risk prediction or prognosis) *Causal inference:* Establish (near) causal effect association between a covariate and an outcome in a given population Estimation of effect size of an intervention on a given outcome in a given patient population	Publication bias Framing Relevance of outcomes	Searching and Aggregating all existing knowledge toavoid unnecessary duplication or indicate gaps in knowledge Prediction of mode of action of different molecular structures as derived from existing knowledge Trial emulation Trial simulation Causal inference techniques Dynamic Decision Problems
Efficacy	Randomized controlled trial	*Causal Inference:* Estimation of effect size of an intervention on a given outcome in a given patient population	Information overload Internal validity (classic bias) Publication bias Incorrect analytical approach Framing Relevance of outcomes Rare side effects not captured in RCTs Transportability/generalizability For static decision problems only	Searching and Aggregating all existing knowledge to avoid unnecessary duplication or indicate gaps in knowledge Causal inference techniques to determine transportability to other patient populations Dynamic Decision Problems
	Observational trial or registry	*Description:* Describe associations between intervention and outcome Describe effect modifiers of that association Describe safety and association with side effects *Prediction:* Describe prognosis and evolution of conditions, and the modifiers *Causal Inference:* Estimation of effect size of an intervention on a given outcome in a given patient population in settings where RCTs are not possible or not ethical	(Unmeasured) Confounding Publication bias Framing Relevance of outcomes Transportability/generalizability	Trial emulation to explore residual confounding Trial simulation
Effectiveness level 1	Systematic review of randomized controlled trials	*Description:* Identify and aggregate available evidence Judge quantity and quality of available evidence *Causal Inference:* Based on effect size estimators, mean changes in outcomes can be estimated at population level when the intervention is implemented Based on subgroup analysis, change in outcome for subgroups can be estimated when the intervention is implemented	Information overload Publication bias Rare side effects not captured in RCTs	Searching and Aggregating all existing knowledge Trial emulation to explore transportability/generalizibility Pragmatic trial approaches
	Systematic review of observational trials	*Description:* Identify and aggregate available evidence Judge quantity and quality of available evidence Describe associations between intervention and outcomes Describe safety and association with side effects		Searching and Aggregating all existing knowledge Trial emulation to explore confounding

Step	Tool/method	Epistemologic aim	Pitfalls/problems	Big data
**Step 2: Socio-economic evaluation**				
Health technology assessment	Incremental Cost Effectiveness Ratio	Estimate cost of the intervention per quality adjusted life year (QALY) as compared to an alternative intervention		Use wearables and online data collection to estimate QoL impact (patient reported outcomes/experience measures) Searching and Aggregating all existing knowledge on this and alternative interventions Detailed description and granularity of target population to help estimate impact in true society (transportability/generalizability)
	Budget Impact	Estimate total cost on population level of the intervention		Detailed description and granularity of target population to help estimate impact in true society (transportability/generalizability)
	Opportunity Cost	Estimate impact of implementation of this intervention on other interventions in the same or other comparable domain		Detailed description and granularity of target population to help estimate impact in true society (transportability/generalizability) Searching and Aggregating all existing knowledge on this and alternative interventions
Societal evaluation	Quality assessment/improvement	*Description:* Describe interventions and outcomes in different settings Describe effect modifiers and case-mix in different settings *Prediction:* Identify subpopulations where certain interventions have different outcome *Causal Inference:* Estimation of effect size of an intervention on a real world population and conditions	Data collection is resource heavy(cost/labor force) Missing data/Incomplete data Cherry picking	Automatic online registration of relevant parameters to allow quality assessment in a sustainable, reliable, complete and cost-effective way
	Pharmacovigilance (Side effects)	Describe safety and association with side effects	Rare side effects not captured in RCTs Side effects might be different in subpopulations not represented in RCTs	Detailed description and granularity of target population to help estimate impact in true society (transportability/generalizability)
	Patient reported outcome/experience measure (PROM/PREM)	*Description:* Describe interventions and patient-relevant outcomes in different settings *Prediction:* Identify subpopulations where certain interventions have different outcome *Causal Inference:* Estimationw of effect size of an intervention on a real world population and conditions	RCTs not representative for real world practice and not using patient relevant outcomes Differences in implementation might cause differences in outcomes	Automatic online registration of relevant parameters to allow quality assessment in a sustainable, reliable, complete and cost-effective way

As a **first** necessary but not sufficient step, the evaluation requires consideration of any evidence on the **efficacy** of the intervention. **AI and big data** can be of help to mine and digest existing literature and evidence, at a much higher speed and in a more exhaustive manner than is currently possible using human skills. It is crucial that for this evaluation of efficacy, **standardized core outcome sets** are applied [15, 16]. These are sets including *only* outcomes that are: relevant to patients; measurable in an accurate and reliable manner; and discriminative. At the micro-level of the healthcare professional and the patient, justifiable healthcare is in fact an essential element to allow for genuine *shared decision making*. Indeed, justifiable healthcare provides all stakeholders involved with the argumentation and information necessary to decide which intervention has the highest probability to lead to the desired outcome given this specific condition affecting this specific patient and taking into account other available interventions. **Big data and AI** can be of help to present alternative options in a way that both patients and healthcare workers can easily understand, and finetuned for the specific case of the patient [17]. Big data and AI can also be used to emulate clinical trials based on existing routinely collected data [18]. This may enable a more rapid collection of evidence on *longer term outcomes*, including on topics for which randomized controlled trials are deemed unfeasible or unethical [19]. The negative effects of smoking, or the choice between different renal replacement therapies can be typical examples of such a context. Moreover, these methodologies allow for an evaluation of *dynamic problems*, such as the optimal timing of the start of certain treatments, or of the succession of different regimens, such as cancer treatments [20]. This is not feasible with clinical trials due to the myriad of potential combinations. Big data approaches can thus have significant added value next to randomized controlled trials, not only to verify the outcome of randomized trials in real world settings, but also to generate knowledge that would be difficult to obtain with randomized trials [21].

The **second** step in our proposed model is an evaluation from the perspective of **health economics**. At his stage, the *budgetary impact* and *opportunity costs* of the possible interventions that have ‘survived’ the first stage (efficacy evaluation) should be taken into consideration.

Inevitably, choices will have to be made during this evaluation process, so in addition to applying economic criteria, in the **third** step, the **ethical** and **social** aspects of the candidate interventions should be explored and evaluated to assess whether the intervention is acceptable and desirable and to what extent it can be considered to be **a priority** that responds to a true need of (a subgroup of) the population.

The *actual performance or outcomes* of the intervention when applied and implemented in **real world conditions** may of course, for a variety of reasons, differ from what was observed under more controlled conditions. Therefore, it is essential that outcomes of the intervention in the *clinical reality* are monitored, for example by establishing registries. Such feedback loops not only provide opportunities to *benchmark* providers, thereby adding granularity to the information for shared decision making at the individual patient level, but also allow for the evaluation of **effectiveness** (i.e. the true effects) of an intervention as opposed to its *efficacy *(Table 2).

**Table 2:** Pitfalls/problems of big data

*Data quality*	
Completeness of data	
Informative missing data	
Selective	
Representative for the problem at hand	
Robustness of data	
Correctness of data	
Relevance of data	
Representative data for the group at hand	
Granularity of data	
Definitions of data labels	
Not uniform	
Not precise	
Not clear	
Dichotomic/categorized	
*Information overload*	
Too much datapoints	
Too much variables	
Known	
Unknown	
Literature overload	
Fast evolution	
Overspecialized	
*Publication/Reporting bias*	
Non-reporting of data	
Reporting of non-prespecified analyses	
Framing	
Unplanned sub-analysis and post-hoc analysis	
*Inappropriate statistical or methodological approach*	
Confusing causal and associative interpretations	
Confusing statistical vs clinical relevance (the p-value problem)	

## Uses of big data and artificial intelligence for evidence building

### Basic science and development

Current biotechnologies such as the different -omics analyses generate massive data, the so-called Biomedical Big Data (BBD). The results of all these research efforts can be dispersed over a wide range of locations, sources and specialties. AI based analytical tools are essential to sifting through all this information, to detecting patterns, and to giving meaning to the outputs of many techniques currently used in biomedical research, such as next generation sequencing, microbiomes, proteomics, etc. AI based search engines can retrieve and visualize all the available data on certain molecules, thus avoiding duplication of research efforts, find new uses for existing molecules (repurposing) or create new molecules with predefined properties [22, 23]. AI can also help to generate new molecules for prespecified tasks, based on structural or functional similarity with other molecules [24]. IBM Watson for Drug Discovery [25] was developed for this purpose and was launched with great ambitions, although its actual real world performance is considered to be a disappointment by many commentators [26]. The latest reports on the capacity of DeepMind’s AI to help unravel the three dimensional structure of proteins [27] demonstrate the potential, but also the narrow applicability of such AI systems: they are great at clearly circumscribed tasks, but transitioning from in silico or in vitro molecular biology to real world clinical applications remains very challenging. Understanding the molecular basis of disease processes can help identify patients in whom certain therapeutic approaches will not work, for example because they lack receptors for the intervention, or because their pathogenetic process runs through different pathways. Many therefore see AI as the ultimate step towards individual patient focused precision medicine. However, unfortunately, biology is mostly more complex than this, and in addition, virtually all of these deep learning analyses are based on associations and not on causal links. A thorough in-depth understanding of causal pathway analysis is therefore often essential to translate results to clinical practice [8].

### Information and evidence gathering

The current rate of publication is such that it substantially outpaces human capacity to read and assimilate all information [28]. Therefore, objective and systematic methods to search, review, and aggregate published studies are a fundamental aspect of evidence building [29, 36]. Many of the tasks involved are extremely time consuming and human resource intensive: setting up and testing a search strategy for each of the different databases (Ovid, Medline, Pubmed, Central…); exclusion of non-relevant papers based on title and abstract; selecting papers based on in- and exclusion criteria of the clinical question; data extraction and aggregation into the different outcomes of interest; applying risk of bias scores to the individual papers [30]. All these tasks need to be done in duplicate, to avoid errors and bias in interpretation. Moreover, information that is not published in indexed journals, also called grey literature, is difficult and time-consuming to find, yet this literature leads to erroneous conclusions [31]. As a result of their laborious character, systematic reviews sometimes are already outdated at the time of publication. However, as these tasks are repetitive in nature, they are in principle ideally suited for automation. Most of these tasks are classification problems which can be addressed by AI based on natural language processing (NPL). Search engines can help plough through the worldwide web to find grey literature information in abstracts and conference proceedings, and even in the data warehouses of administrative organizations such as the Federal Drug Administration (FDA) or the European Medicines Agency (EMA).

AI systems have been developed for each of these individual tasks or combinations thereof. SWIFT-review [32], for example, can be used during the scoping phase to help formulate questions and identify whether they can reasonably be answered based on the available evidence. It could also be used to assist in updating existing systematic reviews. For example, European Renal Best Practice (ERBP), used the Early Review Organization System technology to facilitate and document the first phases of screening and selection of papers in the context of an international multidisciplinary team [36]. EPPI-reviewer [33], used by Cochrane and NICE, not only provides tools to retrieve, select and document papers based on self-learning algorithms, it can also perform automated text coding and data extraction from full papers. As the use of such AI based systems could speed up turn-around time of systematic reviews, substantially more up-to-date systematic reviews could be undertaken. Furthermore, more databases, including FDA and EMA and grey literature, could be explored for information, resulting in more in-depth analysis while at the same time reducing the impact of publication bias. The latter could easily be reduced even more by matching published work with pre-registered protocols, again a task for which AI is ideally suited [34]. Furthermore, once set up and trained, the AI algorithm could be run on a regular basis and eventually update the evidence as new information emerges over time (so-called ‘living’ systematic review).

Before these AI based systems could gain widespread implementation for systematic evidence review, some hurdles remain to be taken however. Crucially, these systems will need to provide evidence of non-inferiority in comparison with human hand-searched systematic reviews [35], so all stakeholders involved can be confident that the same high standards apply for both. Although in principle evidence review is a time linear process, different teams take different approaches to the different subtasks and their timing in the workflow. The different AI systems currently available [30] also mostly work as separate devices on subtasks of the evidence review process, so they need to be integrated in the workflow of the team. Using AI can substantially alter the line of thought here. For example, all systematic review teams agree that framing the question (the PICO framework [36]) should be the first step before any search or data extraction is done. However, using AI, it would be possible to perform the data extraction automatically already at the moment of publication of a paper, and store the data in a knowledge repository for later analysis when the question arises. The methodological, epistemological and other potential sources of bias of such an approach remain to be investigated. Clearly, in order to be generalizable, an AI system should ideally automate all the tasks between the formulation of a question to the presentation of results.

### Generation of knowledge from routinely collected data

Current paradigms place **randomised controlled trials** (RCTs) at the top of the hierarchy of evidence from which to derive information. However, RCTs have several aspects that make them less suited as sources of evidence under certain conditions. *First*, inherent to the requirement for strict in- and exclusion criteria, RCTs typically investigate single, well defined and isolated interventions in very specific subgroups of the population. Whereas this is a strong advantage with regard to the deduction of causal relations between intervention and observed effect, it also has a downside as it hampers the **external validity** of RCTs [37, 38]. In an era where patients increasingly have multiple underlying comorbidities rather than one single disease, this is a serious obstacle. Moreover, for rare diseases, with treatments tailored specifically to particular individuals, simply not enough comparable patients might be present to set up a sufficiently powered RCT.

*Second*, RCTs are very expensive, hence evidence on the efficacy and safety of products with limited commercial interest is often lacking. Some commentators suggest the use of *pragmatic RCTS*, whereby in- and exclusion criteria are broader and administrative regulation is less strict, thus reducing costs. In most settings, such pragmatic trials de facto are a hybrid between a genuine RCT and a prospective, well designed observational trial in which part of the population is randomized [39].

*Third*, RCTs do not solve the problem of what to do *now*, as their results only become available after a significant delay. For example, the IDEAL trial on the timing of start of dialysis in patients with chronic kidney disease took 10 years from the start of the study to the publication of the results [40]. In addition, it appeared that due to the differences in interpretation of the criteria to start, the trial actually answered a question different from the original question it was randomized for [41]. In the field of cardiology, the question on thrombus aspiration during percutaneous coronary intervention was solved more rapidly based on a large registry trial [39] than with the classical RCT [42], even though the budget for the former was 30 times lower. In this era with a rapid evolution and development of new innovative interventions, the effect size of an intervention thus cannot be timely assessed in RCTs as, by the time the RCT is finished, new interventions have become available [43]. Due to all these factors, knowledge generation in medicine would be incomplete and biased if it would only rely on RCTs. The current system is thus suboptimal from an epistemological, ethical and regulatory perspective, and there is an urgent need for complementary ways to generate evidence *next to* RCTs [21].

**Fig. 2:**
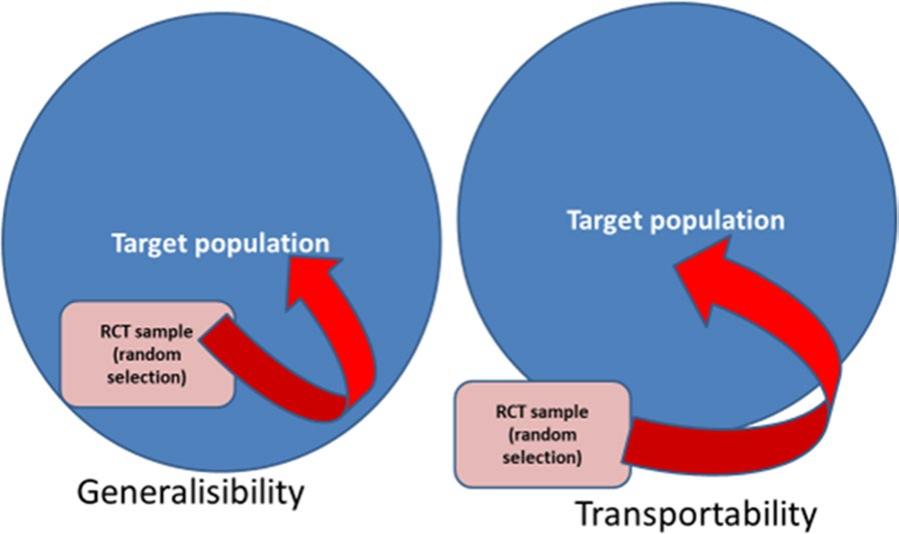
Concepts of generalizability and transportability of data from randomized controlled trials

Besides their use for benchmarking and demonstrating the value of an intervention in real world conditions (see below), routinely collected data can potentially also be used to emulate randomized clinical trials, using an approach known as counterfactual prediction. In this way, routinely collected data can become part of an additional or complimentary methodology to RCTs [21]. Admittedly, existing observational datasets frequently do not contain the necessary granularity to make an exact emulation possible, but about three quarters of trials contain sufficient data to reasonably do so [14]. *First*, using this technique, data from an existing randomized controlled trial can potentially be *generalized* to a broader population. In this way, the validity of the effect size beyond that of the original trial population defined by specific in- and exclusion criteria can be established (Fig. 2). It can also be used to explore *transportability* [44], i.e. to explore the validity of effect sizes in a population that substantially differs from the trial population [45]. Both conditions are becoming increasingly common, as the number of patients with more than one comorbidity is rapidly increasing.

*Second*, the approach of counterfactual prediction potentially enables the simulation of an as yet non-existing randomized clinical trial based on routinely collected data [19]. If such simulation would be successful, the need for expensive RCTs could potentially be avoided, and desired evidence might be obtained more rapidly [39, 42]. Furthermore, RCTs can be considered unethical or simply not feasible in certain settings.

*Third*, the number of potential interventions for any particular condition is growing rapidly, and often these different interventions can be administered in a certain order. For example in HIV or cancer treatment, the succession of different drugs or even types of interventions (radiotherapy, chemotherapy, surgery) will need to be evaluated as these diseases tend to become chronic conditions, requiring different treatments at different points in time (first line vs second line, attack vs maintenance etc.). Also in renal replacement therapy, it is unclear which succession of available treatments (hemodialysis, peritoneal dialysis, transplantation) yields the most optimal outcomes over the life span of the patient [46]. It is nearly impossible to explore all potential combinations and successions of these treatments through RCTs. The optimal *timing* of an intervention, such as starting renal replacement therapy for acute kidney injury, can also be difficult to explore in RCTs. Due to the necessarily strict definitions used in RCTs for deciding the timing of “early” versus “late” start of renal replacement, in the different available RCTs de facto different strategies are compared, explaining differences in outcome. However, it is clear that not all potential definitions of “early” and “late” start can be explored in RCTs.

Such *dynamic problems* could potentially be explored based on routinely collected data, provided that appropriate analytical techniques are used [20]. Emulating RCTs based on routinely collected data could potentially generate evidence in these circumstances, with the advantage that evidence can be updated as new cases come in. Linkage of routinely collected data with RCT data may also leverage RCT findings by enabling increased statistical power, allowing RCTs to be stopped earlier [47], allowing for the identification of relevant effect modifiers, so that treatments can be tailored based on certain markers [48], and earlier validation of surrogate endpoints [49].

### Shared decision making: explaining evidence to patients

**Shared decision-making (SDM)** is increasingly advocated as the preferred conceptual framework for decisions at the individual patient level [50]. The three pillars involved in this decision process are the **evidence base,** the **clinical expertise** of the healthcare worker and the **preferences** and **values** of the patient. The healthcare worker tries to inform the patient on probabilities that intervention A will result in desired outcome X and not an undesired outcome Y, as verbalized by the patient, taking into account the situation of the patient. However, it is essential that the patient and physician can *obtain, understand* and correctly *interpret* the information provided. As both patients [51] and physicians [52] often lack basic statistical literacy, information needs to be presented in a way that helps them gain insight in the data and their meaning in a simple, informative and straightforward way [53, 54].

It is therefore crucial to develop easily **understandable presentation** paradigms that allow for the integration of all available evidence with the specific condition of the individual patient, helping her to make a decision that leads to a result as close as possible to her preferences and values. A healthcare worker has an obligation to first elicit the true values and life goals of the patient before considering a treatment. No interventions should be made to achieve outcomes that are of no value to a given patient, for example interventions that are only intended to optimize surrogate outcomes which are irrelevant to the patient. The proposed treatment should be that which has the highest probability of achieving these values and life goals. Situations may of course arise in which the values of the healthcare worker and those of the patient differ and where, from the perspective of the healthcare worker, a suboptimal decision is made. The shared decision making process is akin to a balanced negotiation process wherein both parties try to achieve the best decision. The stronger the evidence (for example several large randomized controlled trials with similar results), and the more important the outcome (for example an important improvement in survival), the greater the effort the healthcare worker must make to convince the patient. Graphical representations of the projected outcome of different treatment alternatives can be constructed in real time by algorithms based on routinely collected data, containing features similar to the target patient, but treated with the different alternative treatments available [17]. Such real time visualizations of the different options can be used to help achieve the goal of genuinely shared decision making [17, 53].

### The need for core outcome sets

If we intend to use aggregated evidence from randomized trials and observational studies to assess outcomes that are relevant for patients, it is essential to create standardized core outcome sets [15]. A core outcome set is a compilation of well-defined outcome domains relevant to patients, with a unified, well circumscribed definition of the measure used to evaluate the outcome domain, and the desired way to report it. The *unique* definition is essential to allow for aggregation of data across studies and to ensure that each study reports on the same construct of the outcome. Currently, many outcomes are ill-defined and have different meaning in different studies, leading to misinterpretation and confusion [55]. Even when there is a unified definition, the interpretation and application of this definition should be as uniform as possible. Differences in what is reported as an outcome substantially limit the progress of knowledge and make the aggregation of evidence difficult if not impossible, as such differences result in comparing apples and oranges. Moreover, using standardized outcomes will decrease research waste, as studies will only investigate those outcomes that are relevant to patients and society. There is a growing understanding of the importance of this problem by scientific organisations [56–58]. Some administrative and commercial initiatives have also been launched in this regard, e.g. ICHOM (International Collaborative on Health care Outcome Measures) [59].

Acute kidney injury is a clear example. For a long time, a unified definition was lacking, leading to a wide divergence in reported incidence, prevalence and outcomes [10]. Over the last years, a unified definition has been formulated and accepted [60], yet the practical interpretation and implementation of this “unified” definition still is open to interpretation [61], with substantial impact on reported incidence, prevalence and outcome of AKI [62]. When data are aggregated in big data sets, it is essential that the constructs that are represented by those data are defined and measured as uniformly as possible. If not, the “tank problem” [63] might arise, i.e. patients are categorized based on criteria that are not linked to their underlying pathology, but to some other, mostly technical aspect. This has been found, for example, with regard to the automated diagnosis of pneumonia, where diagnosis was strongly influenced by the type of X-ray device used for imaging [64].

To be useful in Shared Decision Making, standardized outcomes should also be *relevant* for all stakeholders [16]. This means that patients should be involved in constructing and selecting the outcome variables of interest, how they should be measured and which difference in that outcome is relevant to them [65]. The need for standardized outcomes will be further exacerbated if we start using AI to explore evidence. AI will either used predefined terminology, so it can search for predefined terms, or natural language processing to extract concepts from existing texts. In both cases, standardization of the outcomes is essential. If AI uses predefined concepts in its search, we need to ensure that these concepts all have the same meaning in the primary sources, for otherwise information will be lost and may even be wrong, as an apple might not always truly be an apple. If we are going to use natural language processing, there are strong reasons to be concerned that AI will get stuck in the understanding the true meaning of expressions it encounters during the text analysis and in placing them in a general context, a task that can easily be performed by humans, but is very hard for AI.

#### The use of big data in health economics analysis (affordability and prioritization)

Over the last decades, healthcare expenses have surged exponentially. Although partially explained by the ageing of the population, the bulk of this increase can be attributed to a steep increase of technological interventions, both in terms of availability and accessibility, as well as to the cost of these interventions. It is obvious that there is a limit to the total budget that can be spent on healthcare, which inherently implies that choices need to be made all the time. To ensure that these choices result in justifiable healthcare, a thorough analysis is necessary. *First*, an assessment of the cost of the intervention in relation to its potential impact needs to be performed. The indicator of “*quality adjusted life years gained*” (QALY) is most frequently used in this context. The utility of an intervention is based on available evidence regarding the estimated effect size, with systematic reviews being the ideal instrument to calculate these. As mentioned earlier, big data and AI can be used to help perform such systematic reviews if they are lacking.

*Second*, the budgetary impact of the intervention needs to be assessed, i.e. the cost of the intervention times the number of persons in the society who would potentially benefit from that intervention. Registry data can be used to assess these. In nephrology, for example, registries could establish the number of people with diabetes mellitus type 2 and micro- or macro-albuminuria, thus also assessing the number of people who could potentially benefit from a drug that retards progression of kidney disease. Ideally, data on the degrees of comorbidities in this population should also be available, to assess the extent to which available evidence can be generalized to this specific real-world population, in order to estimate the true effect and thus the real expected QALYs, as described above.

Evidence from trials and registries could potentially be complemented by real world data from wearables, handheld devices and social media to help assess the utility of interventions in everyday living conditions of patients [66]. Many more databases pertaining to people with different backgrounds are needed for health economic analysis than in the case of effect size estimation. For example, in order to estimate costs, not only data on the incidence and prevalence of disease conditions and data regarding costs are needed, but also information on the extent of the associated comorbidities and their distributions.

Given that all of these data need to be integrated, the use of big data for the purposes of health economics analysis might be jeopardized even more by concerns on data management and data quality in the data sources and the analytical techniques used [67]. Data management issues, such as data storage, computation power, opaque access, integration and linkage of datasets, and ensuring the uniqueness of used definitions, can be mitigated by creating standardized approaches to storage and definitions of data. Still then, the cost of creating and/or accessing all these datasets might be prohibitive for health economists to access and integrate all the databases they ideally would need to feed their models. Most machine learning algorithms are developed for prediction based on associations, for which they perform quite well. However, prediction is quite different from estimating the effect sizes of interventions, where it is essential that the relation between variables and outcome is *causal*. When applying machine learning in comparative health economics, it is moreover essential that the algorithms can handle the data in a counterfactual way: what would have happened if, instead of intervention A, intervention B would have been implemented? [68].

It is an open question whether official bodies should accept observational data, even when “big”, as a substitute for randomized controlled trials [69]. Nevertheless, the US and European drug regulators (cf. the Twenty-first Century Cures Act in the US and the Adaptive Licensing approach in the EU) propose that in some cases Phase III trials could be replaced by post-marketing evidence based on routinely collected data studies [70]. This paradigm shift not only entails ethical and regulatory challenges, but also substantial methodological challenges because drawing valid causal conclusions from routinely collected data necessarily relies upon crucial assumptions about the causal structure of the real world beyond what is encoded in the data. This fundamentally changes the paradigm of the safety and effectiveness assessment from a process with clearly distinctive phases to a continuous process in which post-marketing evidence derived from routinely collected data plays an important role [71].

Likewise, for **medical devices**, the new **Medical Device Regulation** (MDR) (Regulation (EU) 2017/745) in the EU indicates an evolution towards the increased importance of post-marketing surveillance based on routinely collected data. Increased use of routinely collected data could provide valuable information on safety and effectiveness but the credibility, transparency and enforceability of their role in post-market surveillance should be explored and demonstrated [72–75]. Various remaining regulatory uncertainties, for example regarding the need to make public whether or not post-approval studies have begun, or the timing of confirmatory clinical trials, have spurred criticisms that such procedures might progressively lead to de-regulation [70, 72, 74].

#### The use of big data for safety and benchmarking

##### Safety monitoring and surveillance outcomes of interventions

According to some commentators, systems based on available routinely collected data can potentially accommodate for some of the limitations of monitoring based on spontaneous reporting, considered as a cornerstone so far [76], such as the underreporting of non-obvious side effects. Several pilot programs in the US (e.g. OMOP and the Sentinel initiatives), the EU (e.g. EU-ADR and PROTECT) and Asia (e.g. Asian Pharmacoepidemiology Network or AsPEN) assess the potential of routinely collected data for pharmacovigilance and routine signal detection. However, partly due to limited statistical standards for risk assessment, none of these initiatives has convincingly provided credible or reproducible evidence of unexpected adverse drug reaction or confirmation of known harms [73]. A literature review has compared a broad range of analytical approaches and identified traditional pharmaco-epidemiological designs (in particular, self-controlled designs) and sequence symmetry analysis as two of the most promising approaches for signal detection in routinely collected data [76]. An outcome-wide approach [77] to pharmaco-epidemiological designs based on propensity score analysis may considerably reduce modelling and computational demands, thereby increasing their suitability for routine signal detection, with a minimal risk of bias.

##### Benchmarking and effectiveness

Although RCTs continue to rank high in the pyramid of evidence, they suffer from some inherent problems such as a lack of generalizability and transportability, as discussed above. In addition, for some interventions, the *way in which RCTs are implemented* in real clinical practice could substantially impact the difference between the effect size in real life and that the effect size observed in RCTs, where conditions are mostly optimal. For technical interventions such as surgical operations, catheterisations, and diagnostic procedures, the skill and expertise of the operator can have a substantial impact on the final outcome and will determine whether the results can be replicated or not. If the operator has much skill and experience with intervention B, in her hands the outcome with this intervention B could result in better outcomes than with intervention A, even if in a randomized trial where A was applied by operators skilled in A, the latter was superior.

Currently, the quality of delivered healthcare is mostly the result of team work and of a succession of events, from correct referral over correct diagnostic procedure and interpretation, to correct identification and attention to safety and a culture to avoid accidents, including basic nursing care. Hence it is not only the individual skill of an individual operator or one single intervention that will determine the final result, but rather the full chain of all processes and people involved in the total process. Even for simple interventions, such as the dosing of dialysis for acute kidney injury, differences in outcomes between RCTs can be explained by differences in overall practice between centres [78]. Typically, with studies done in the setting of a single centre, exceptional attention is given to all study participants, as the team believes in the investigated treatment, which is far less so in multicentric trials. The routine collection of outcome data offers opportunities to evaluate and illustrate the performance of healthcare providers at both the micro level of the individual provider and the meso level of the organization. The technical possibilities of Big Data approaches allow for collection of the data necessary to produce such evaluations from different sources and turning them to a meaningful construct. For example, the outcome of a cancer intervention can be assessed by accessing laboratory, pathology or radiology data warehouses to collect data from an individual diagnosed with the cancer, and linking them with the persons involved in the care as well as to other outcomes such as mortality, medical costs, social welfare and employment, need for societal support and other parameters derived from various other available datasets.

From all these data, algorithms can derive different markers of performance. The latter can subsequently be used as feedback to the healthcare workers (formative evaluation), or to inform patients on the performance of different healthcare institutions/providers in domains that might be of interest and value to them. In this way, Big Data could contribute to value based healthcare [79] and shared decision making. Whereas the strict technological requirements to assemble such online repositories will probably be resolved in the near future, some more fundamental methodological questions remain to be answered before such systems can be safely and effectively used in clinical practice. The selection of the most relevant constructs to reflect “performance” has been discussed already (cf. supra) and should follow the same procedures as those for establishing standardized core outcome sets. Furthermore, it should be questioned how the feedback to healthcare professionals should be structured and organized in order to to achieve a true improvement in the quality of care provided. Currently, most of these systems use a benchmarking against a mean. However, there is evidence that follow up of performance of an individual over time or comparison with accepted and established criteria might be much more effective to induce a positive change in behaviour [80]. Finally, one should be careful when designing the presentation of data to patients, as they can struggle with interpreting the information offered [17].

##### Patient reported outcomes/patient reported experiences

Patient perspectives and experiences are increasingly gaining interest. Patient-reported outcome measures (PROMs) and experience measures (PREMs) are mostly questionnaires that assess patients’ health, health-related quality of life and other health-related constructs. They can be used to evaluate performance or as a benchmark to inform patient choice for healthcare. However, when used intelligently, the information can also be used to discover unmet needs or preferences in the approach and management of certain health conditions or patient groups, assess the effectiveness of different treatment plans, monitor disease progression, stimulate better communication, promote shared decision making and issue tailored advice and education [81–83]. PROMS and PREMS allow for the visualization of the outcome of certain interventions in some of the real treatment centres available in the region of the patient rather than results obtained in the highly controlled setting of an RCT.

Patient reported outcomes and experiences mostly are collected as a one-off (cross-sectional) assessment, most frequently using pencil and paper, resulting in a burden for patient and staff. As a consequence, surveys are restricted in size, decreasing the relevance and spectrum of the topics explored. The advent of new digital technologies opens the door to more continuous, in-depth and online reporting of symptoms and experiences of patients, in a more feasible, sustainable and cost-effective way [84]. In the most simple format, patients can use a tablet or handheld device to complete questionnaires during waiting times in the hospital. More sophisticated systems allow for a continuous reporting of symptoms and outcomes through smartphones or wearables. Some systems rely on algorithms that infer treatment recommendations or advice to plan earlier consultations from the data provided [85]. Such systems for digital symptom reporting can have a positive impact on the quality of healthcare with reduced symptom distress, improved symptom burden through better self-management, improved health-related quality of life and higher quality of interaction with healthcare professionals.

Going one step further would be to track patient data continuously, for example by using smartwatches to register heartbeat, or geolocation to assess mobility, activity and independence as a surrogate of well-being [66]. Even ‘smart’ pills, monitoring adherence to medication intake, are possible these days using AI technology [86]. Although attractive at first sight, many unresolved issues remain before such systems can be more widely used [86]. Major points of concern are the safeguarding of privacy and the likely impact on attitudes of insurance companies and healthcare organizations when the possibility of eavesdropping on all movements of patients all of the time makes it easier to distinguish high and low risk patients from their point of view.

## Conclusions

Over the last decades, the face of health care has changed dramatically. New technologies and interventions are being developed at exponential speed. At the same time, the type of health problems has shifted from patients with a clearly defined single and mostly acute condition to patients with progressively more comorbidity and chronic diseases. Assessment of the effectiveness and efficacy of interventions in such a setting becomes more difficult, and requires the evidence derived from randomised controlled trials to be complemented with evidence derived from routinely collected data. However, we need to ensure that the right methodological approaches are used and that data curation is done with utmost attention to quality.

New interventions also put additional pressure on an already spiralling health care budget. Therefore, not only the effectiveness of an intervention, but also the social impact and the overall budgetary consequences should be taken into account. To enhance patient centredness of health care, tools are needed to support shared decision making. Ideally, this should be done using data from the centre where the patient will be treated, and it should be based on outcomes in patients as similar as possible to the target patient. Here, too, big data and AI could potentially be helpful to explain and visualize the effect of different interventions to patients. In this paper, we have proposed a novel framework to help evaluate whether interventions will result in justifiable healthcare, i.e. healthcare that is efficacious, fair, equitable and sustainable. We have identified where big data and AI could potentially be helpful in this evaluation. Further research is needed to explore the epistemological, legal and ethical challenges of the use of big data and AI within this framework.

## Data Availability

Not applicable.

## Abbreviations

AI: Artificial intelligence
AKI: Acute kidney injury
EMA: European Medicines Agency
ERBP: European Renal Best Practice
FDA: Federal Drug Administration
PICO: Patient Intervention Comparator Outcome
QALY: Quality adjusted life year
RCD: Routinely collected data
RCT: Randomised controlled trial
RWD: Real world data
SDM: Shared decision making
